# Impact of the COVID-19 Pandemic on Colorectal Cancer Surgery: Surgical Outcomes and Tumor Characteristics in a Multicenter Retrospective Cohort

**DOI:** 10.3390/jcm14196732

**Published:** 2025-09-24

**Authors:** Emrah Sahin, Sami Akbulut, Zeki Ogut, Serkan Yilmaz, Yasin Dalda, Adem Tuncer, Zeynep Kucukakcali

**Affiliations:** 1 Department of Surgery and Liver Transplant Institute, Inonu University Faculty of Medicine, 44280 Malatya, Turkey; 2 Department of Biostatistics and Medical Informatics, Inonu University Faculty of Medicine, 44280 Malatya, Turkey; 3 Department of Surgery, Elazığ Fethi Sekin City Hospital, 23300 Elazig, Turkey; 4 Department of Surgery, Firat University Faculty of Medicine, 23119 Elazig, Turkey

**Keywords:** COVID-19 pandemic, colorectal cancer, emergency surgery, tumor characteristics, surgical outcomes

## Abstract

**Background:** Colorectal cancer (CRC) is a leading cause of cancer-related mortality worldwide. The COVID-19 pandemic disrupted healthcare systems globally, raising concerns about delays in CRC diagnosis and treatment, and their potential negative effects on surgical outcomes. However, the extent of this impact remains uncertain. **Aim:** To compare the clinical characteristics, treatment strategies, and outcomes of CRC patients between the Pre-COVID-19 and COVID-19 Era groups, and to identify independent predictors of metastasis and mortality. **Methods:** This retrospective multicenter study included 397 CRC patients who underwent surgical treatment between 1 July 2018, and 1 August 2021, at three tertiary medical centers. Patients were divided into two groups: Pre-COVID-19 (n = 213) and COVID-19 Era (n = 184). Demographic data, tumor characteristics, surgical approach, postoperative complications, and survival outcomes were analyzed. Logistic regression analysis was conducted to identify independent predictors of metastasis and mortality. **Results:** The median age was 64 years (95% CI: 63–66), with 59.2% being male. Compared to the Pre-COVID-19 group, patients in the COVID-19 Era had significantly larger tumors (*p* < 0.001), with a significantly higher total LN retrieved (*p* = 0.006), more advanced T-stage (*p* = 0.007), higher N2 lymph node involvement (*p* = 0.027), and poorer tumor differentiation (*p* = 0.030). Intestinal perforation was more frequent in the Pre-COVID-19 group (*p* = 0.042). Multivariate analysis revealed increased odds of mortality associated with the positive LN retrieved (OR: 1.14; *p* = 0.001), moderate tumor differentiation (OR: 2.99; *p* = 0.043), poor differentiation (OR: 4.57; *p* = 0.023), undifferentiated histology (OR: 6.95; *p* = 0.028), intestinal obstruction (OR: 2.67; *p* = 0.007), intestinal perforation (OR: 11.76; *p* < 0.001), and distant metastasis (OR: 2.86; *p* = 0.008). Regarding metastasis, elevated preoperative CEA (OR: 1.02; *p* = 0.002), lymph node involvement (OR: 4.87; *p* = 0.002), and perineural invasion (OR: 2.17; *p* = 0.033) were independently associated with increased odds of metastasis. **Conclusions:** Although overall survival did not differ significantly between groups, patients treated during the COVID-19 Era exhibited more advanced histopathological characteristics, including a higher proportion of T4 tumors, increased N2 lymph node involvement, and poorer differentiation grades. Despite no significant differences in postoperative complications between groups, it is noteworthy that preoperative intestinal perforation was less frequent in the COVID-19 Era cohort.

## 1. Introduction

Severe Acute Respiratory Syndrome Coronavirus-2 (SARS-CoV-2), the novel betacoronavirus responsible for coronavirus disease 2019 (COVID-19), was first identified in December 2019 in Wuhan, Hubei Province, China, following a cluster of atypical pneumonia cases epidemiologically linked to a seafood wholesale market [1,2,3,4,5]. The rapid geographic spread of the virus, combined with its potential for severe respiratory illness, multi-organ involvement, and high transmissibility, prompted the World Health Organization (WHO) to declare the outbreak a Public Health Emergency of International Concern (PHEIC) on 30 January 2020 [6]. On 11 March 2020, which coincides with the date of the first confirmed COVID-19 case in Turkey, the WHO officially characterized COVID-19 as a global pandemic, marking the first such declaration since the 2009 H1N1 influenza outbreak [1,2,3,4]. By end of November 2024, over 776 million confirmed cases and more than 7.1 million attributable deaths had been documented globally [6,7]. Epidemiological indicators underscore pronounced regional heterogeneity in transmission dynamics, disease burden, and health system responsiveness, highlighting the sustained and profound impact of the pandemic on global health infrastructure [7]. The ensuing health crisis imposed unprecedented strain on healthcare delivery worldwide, precipitating large-scale reallocation of medical resources and widespread disruption of non-COVID-19 health services [8,9,10].

Multiple epidemiological studies have reported that cancer screening rates declined by approximately 0% to 77% in various countries [11,12,13], with some regions experiencing even greater reductions due to prolonged strain on healthcare systems. Prior to the COVID-19 pandemic, colorectal cancer (CRC) screening programs were instrumental in facilitating early detection and enabling timely surgical intervention. However, the pandemic-driven reallocation of medical resources led to a marked reduction in screening uptake and prolonged delays in treatment initiation. Elective surgical procedures were deferred by as much as 12 weeks, heightening concerns over potential disease progression [13,14]. Among the healthcare domains most adversely affected, oncological care—particularly the management of CRC—faced substantial challenges, including postponement of curative-intent treatments and deterioration in oncological outcomes [15,16,17].

According to GLOBOCAN databases, CRC remains a major global health concern, with an estimated 1.9 million new cases and over 900,000 deaths annually, marking a steady rise in incidence compared to previous years [18,19,20]. It is the third most commonly diagnosed cancer and the second leading cause of cancer-related mortality [19,21,22,23]. The incidence of CRC continues to rise, particularly in developing countries where access to screening and early detection programs remains limited [24]. In contrast, high-income countries have seen stable or declining incidence rates due to improved screening programs [14], though an aging population and lifestyle factors continue to contribute to the overall burden [25]. These disparities highlight the need for global efforts to enhance CRC prevention, early diagnosis, and equitable access to treatment [25].

The timely diagnosis and surgical management of CRC are critical for improving survival rates, postoperative recurrence and minimizing complications [26,27]. However, during the COVID-19 pandemic, healthcare resources were reallocated, elective procedures were postponed, and routine cancer screenings declined significantly [8,9,10,23]. Studies indicate that CRC screenings dropped by 38.4% to 84.5% in various regions [28,29,30], resulting in a substantial increase in Stage III and IV CRC cases, which negatively impacted overall survival rates [16,23]. Additionally, reductions in colonoscopy procedures and delays in referrals exacerbated disease progression risks [31,32]. These disruptions raised concerns regarding increased tumor burden at diagnosis, worsening oncological outcomes, and higher postoperative complications [33,34].

Despite these challenges, the long-term consequences of pandemic-related delays on CRC treatment outcomes remain uncertain, with ongoing studies attempting to quantify their full impact [31]. Understanding these disruptions is crucial for developing more resilient healthcare systems, ensuring uninterrupted CRC treatment during future crises, and improving global cancer care strategies. Some multicentric analyses suggest that while there were disruptions in diagnostic pathways, surgical timing remained stable in certain high-volume centers [8]. However, tumor characteristics such as increased lymph node involvement and larger tumor sizes suggest an indirect effect of the pandemic on CRC progression [35]. Understanding these epidemiological trends is essential for shaping future health policies and ensuring resilience in oncological care during global crises [17].

This multicentric study primarily aims to investigate how the COVID-19 pandemic influenced CRC surgical management, focusing on treatment timelines, tumor characteristics, and oncological outcomes. It specifically evaluates whether pandemic-related delays in diagnosis and treatment modified disease presentation or postoperative prognosis. Drawing on data from three high-volume centers, the study compares variations in surgical timing and pathological findings, aiming to provide evidence-based insights into healthcare system adaptability and to guide optimization of CRC care during future health crises. As a secondary objective, the study performed multivariable analyses—considering COVID-19 status as an independent variable—to identify independent predictors of mortality and metastasis in colorectal cancer, irrespective of the pandemic period. 

## 2. Materials and Methods

### 2.1. Type, Duration, and Location of the Study

This retrospective case–control study includes patients who underwent CRC surgery between 1 July 2018, and 1 August 2021. This multicenter study was conducted across three centers: Inonu University, Fırat University, and Fethi Sekin City Hospital, where patients underwent surgery in the General Surgery Departments, ensuring a diverse patient population and comprehensive data collection.

### 2.2. Determination of the Study Group

Based on data from the patient information and management system utilized in three hospitals participating in this retrospective study, a total of 397 patients who underwent CRC surgery between 1 July 2018, and 1 August 2021, were included. Of these, 213 patients who received surgical treatment between 1 July 2018, and 10 March 2020, were categorized as the Pre-COVID-19 group. The remaining 184 patients who underwent CRC surgery between 11 March 2020, and 1 August 2021, were classified as the COVID-19 Era group.

### 2.3. Inclusion and Exclusion Criteria

This study included CRC patients who underwent surgical treatment at the three aforementioned centers and whose postoperative follow-up and treatment continued at these institutions. Patients diagnosed with CRC but not operated on, those who underwent surgery at other centers and only received oncological treatment at the three included institutions, or those referred to the pathology departments of these centers solely for histopathological evaluation were excluded from the study. Additionally, patients with insufficient data for statistical analysis were also excluded from the study.

### 2.4. Definitions, Parameters, and Variables Used in the Study

Data from the three institutions were retrospectively retrieved from hospital records and compiled into Excel. Demographic characteristics, surgical details, histopathology, and postoperative follow-up were recorded. The ‘’diagnosis-to-surgery‘’ period referred to the time from biopsy report to surgery, while the ‘’surgery-to-pathology report‘’ period was from surgery to the final pathology report. For survivors, follow-up duration was from surgery to the last outpatient visit or phone contact; for deceased patients, from surgery to date of death [2]. These standardized definitions allowed evaluation of diagnostic and reporting delays during the pandemic.

Neoadjuvant therapy was indicated for locally advanced CRC per TNM criteria, especially in rectal cancer with clinical T3–T4 or clinical node positive disease [36]. High-risk features included mesorectal fascia involvement, extramural venous invasion, or suspected lateral pelvic lymph node metastases. Total neoadjuvant therapy indications included clinical T3c/d–T4, clinical positive node 2, or positive extramural venous invasion [37]. Decisions were based on high-resolution pelvic magnetic resonance imaging, multidisciplinary discussion, and consensus guidelines.

Adjuvant therapy was recommended for stage III CRC, and selectively for high-risk stage II disease [38]. High-risk features prompting adjuvant therapy in proficient mismatch repair patients included: <12 examined lymph nodes, vascular/lymphatic/perineural invasion, tumor perforation, obstruction, high tumor budding (≥10 buds), elevated preoperative CEA, close/positive margins, poor differentiation (signet ring, mucinous, undifferentiated), or T4 invasion of visceral peritoneum/adjacent structures [39,40]. Tumor staging followed the American Joint Committee on Cancer (AJCC) Cancer Staging Manual, 8th Edition, utilizing the Tumor-Node-Metastasis (TNM) classification system as the standard reference for colorectal cancer staging [41].

Variables compared included: age, gender, comorbidity (hypertension, diabetes mellitus, cardiac, pulmonary, thyroid disorders), American Society of Anesthesiologists (ASA) score, tumor location (right colon, left colon, rectum, sigmoid, cecum, rectosigmoid, transverse colon), timing of surgery (elective, emergency), preoperative conditions (intestinal obstruction, intestinal perforation), type of surgery (open, laparoscopic, conversion), presence of ostomy, mucinous component, tumor differentiation (well, moderately, poorly differentiated, undifferentiated), TNM classification (T: primary tumor size, N: nodal involvement, M: metastasis), perineural invasion, lymphovascular invasion, overall postoperative complications, specific postoperative complications (intestinal obstruction, anastomotic leak, intra-abdominal abscess, pulmonary complications, wound infection, sepsis, metabolic complications), neoadjuvant therapy, adjuvant therapy, and patients outcomes (survivors, non-survivors).

### 2.5. Study Protocol and Ethics Committee Approval

The study adhered to the Declaration of Helsinki and institutional/national ethical standards. Formal approval was granted by the Inonu University Institutional Review Board (IRB) for non-interventional studies (Approval No: 7439; Date of Approval: 25 March 2025). The study followed the Strengthening the Reporting of Observational Studies in Epidemiology (STROBE) guidelines to enhance methodological transparency and reproducibility [42].

### 2.6. Statistical Analysis

The study variables were analyzed using absolute and relative frequencies for categorical data and median values with a 95% confidence interval (CI) and interquartile range (IQR: Q1–Q3) for numerical data. Normality of data distribution was assessed using the Kolmogorov–Smirnov test. Non-parametric statistical tests were applied when data deviated from a normal distribution. Statistical tests were selected based on the type and distribution of variables. The Mann–Whitney U test was utilized for comparisons between two independent groups, while Fisher’s exact chi-square test, chi-square test with Yates correction, and Pearson chi-square test were used to assess associations between categorical variables where applicable. Kaplan–Meier survival analysis was conducted to evaluate the impact of mortality status and the Pre-COVID-19 and COVID-19 Era groups on survival outcomes. Survival rate differences were compared using the log-rank test. Logistic regression analysis was performed to comprehensively evaluate predictors of both metastasis and mortality in CRC. In this model, COVID-19 status was included as a variable, but the primary goal of the analysis was to identify additional independent risk factors beyond the pandemic context, providing insights into determinants of outcome irrespective of era. Variables with a *p*-value < 0.05 in univariate analysis and clinically relevant variables were included in the logistic regression model. The model’s goodness of fit was assessed using the Hosmer–Lemeshow test. A *p*-value < 0.05 was considered statistically significant. All statistical analyses were performed using IBM SPSS Statistics for Windows, Version 25.0 (IBM Corp., Armonk, NY, USA).

## 3. Results

### 3.1. Overall Characteristics

Table 1 and Table 2 present the continuous and categorical variables of the entire study cohort. A total of 397 patients diagnosed with CRC were included in this study. In this cohort of patients who underwent surgery for CRC, the median age was 64 years (IQR: 53–73; 95% CI: 63–66), reflecting a predominance of older adults. The median interval from diagnosis to surgery was 8 days (IQR: 5–14; 95% CI: 8–10), while the median time from surgery to receipt of pathology results was 22 days (IQR: 14–34; 95% CI: 21–25). Preoperative tumor marker levels demonstrated a median CA19-9 of 22.5 U/mL (IQR: 8–125; 95% CI: 18–34) and a median CEA of 2 ng/mL (IQR: 1–7; 95% CI: 2–3). The median tumor size measured 45 mm (IQR: 33–60; 95% CI: 45–50). Lymph node assessment revealed a median of 23 total lymph nodes retrieved (IQR: 14–38; 95% CI: 22–26), with a median of positive lymph node (IQR: 1–3; 95% CI: 1–2). The median hospital stay was 9 days (IQR: 7–13; 95% CI: 9–10), and the median follow-up duration was 49 months (IQR: 38–59; 95% CI: 47–51), underscoring the postoperative monitoring in this entire study population, with an approximate follow-up period of 5 years.

A total of 397 patients with CRC were included in the study. Of these, 53.7% underwent surgery before the COVID-19 pandemic and 46.4% during the COVID era. The majority of cases were treated at Inonu University (52.9%; n = 210), followed by Firat University (29.7%; n = 118) and Elazig City Hospital (17.4%; n = 69). Males comprised 59.2% (n = 235) of the study population, and overall comorbidity was present in 47.9% (n = 190) of CRC patients. Specifically, diabetes mellitus was present in 20.2%, hypertension in 25.7%, pulmonary disease in 9.6%, cardiac disease in 16.6%, and thyroid disorders in 8.1% of cases. According to ASA classification, 5.0% were ASA I, 42.1% ASA II, 51.1% ASA III, and 1.8% ASA IV.

The most common tumor location was the rectum (34.5%), followed by the right colon (21.4%), sigmoid colon (19.9%), cecum (10.1%), left colon (8.1%), rectosigmoid (4.5%), and transverse colon (1.5%). Elective surgery was performed in 75.8% of patients, whereas 24.2% required emergency intervention. Preoperative intestinal obstruction was recorded in 19.4%, and perforation in 4.8% of cases. Open surgery was the predominant approach (51.6%), with laparoscopic surgery in 44.6% and conversion to open in 3.8%. Ostomy formation during the index surgery occurred in 48.9% of patients.

Histopathologically, a mucinous component was present in 12.9% (n = 50) of tumors. Tumor differentiation was well in 21.0%, moderate in 64.0%, poor in 12.4%, and undifferentiated in 2.6% of cases. Primary tumor staging revealed T1 in 3.8%, T2 in 12.0%, T3 in 58.7%, T4 in 24.5%, and Tis in 1.0% of patients. Lymph node involvement was absent (N0) in 49.6%, while N1, N2, and N3 disease were observed in 28.0%, 22.1%, and 0.3%, respectively. Distant metastasis (M1) was present in 20.2% of cases. Perineural invasion was noted in 36.9% and lymphovascular invasion in 60.2% of tumors.

Postoperatively, 31.0% of CRC patients experienced complications, with intestinal obstruction (5.5%), anastomotic leak (3.0%), intra-abdominal abscess (4.5%), wound infection (12.9%), sepsis (5.3%), pulmonary complications (5.5%), and metabolic complications (3.5%) being the most common. Neoadjuvant therapy was administered in 17.1% of patients, adjuvant chemotherapy in 68.6%, and adjuvant radiotherapy in 10.8%. At a median follow-up of 49 months, 75.1% of the entire cohort were surviving (n = 298), while 24.9% were non-surviving (n = 99).

### 3.2. Pre-COVID-19 Vs. COVID-19 Era Groups

#### 3.2.1. Demographic and Clinical Characteristics

Table 3 and Table 4 present a comparative analysis between patients who underwent CRC surgery during the Pre-COVID-19 period and those treated during the COVID-19 Era. The median age was 63 years (95% CI: 62–65) in the Pre-COVID-19 group and 65 years (95% CI: 63–69) in the COVID-19 Era (*p* = 0.579). Gender distribution was similar between groups (male: 60.1% vs. 58.2%, *p* = 0.695). Overall comorbidity was slightly less frequent in the COVID-19 Era (45.1% vs. 50.2%, *p* = 0.308). The rates of diabetes mellitus (21.2% vs. 19.3%, *p* = 0.630) and hypertension (24.5% vs. 26.8%, *p* = 0.600) were comparable.

#### 3.2.2. Tumor Characteristics and Stage

Median tumor size was significantly larger in the COVID-19 Era group (50 mm, 95% CI: 50–58) compared to the Pre-COVID-19 group (42 mm, 95% CI: 40–45) (*p* < 0.001). The proportion of T4 tumors was higher in the COVID-19 Era (31.9% vs. 18.1%, *p* = 0.007), as was lymph node (N2) involvement (28.4% vs. 16.7%, *p* = 0.027). Distant metastases (M1) were more frequent during the pandemic period (23.4% vs. 17.4%, *p* = 0.137). High-risk histopathological features such as perineural invasion (45.3% vs. 29.7%, *p* = 0.001) were significantly more prevalent in the COVID-19 era group, while lymphovascular invasion showed no significant difference (62.6% vs. 58.1%, *p* = 0.360).

#### 3.2.3. Surgical and Therapeutic Approaches 

Elective surgery rates were slightly higher during the COVID-19 Era (78.3% vs. 73.7%, *p* = 0.291). Open surgeries increased (56.5% vs. 47.4%, *p* = 0.179) and laparoscopic surgeries decreased (39.7% vs. 48.8%). Neoadjuvant therapy was used less frequently during the pandemic (13.3% vs. 20.5%, *p* = 0.081), while adjuvant chemotherapy rates were similar (67.4% vs. 69.5%, *p* = 0.661).

#### 3.2.4. Postoperative Outcomes

Overall postoperative complication rates were slightly higher in the COVID-19 Era (33.1% vs. 29.1%, *p* = 0.385), with minor increases in intestinal obstruction (7.1% vs. 4.2%), anastomotic leak (3.8% vs. 2.4%), and intra-abdominal abscess (3.8% vs. 5.2%). Hospital stay was similar between groups (8 vs. 9 days, *p* = 0.422). The incidence of intestinal perforation was significantly lower in the COVID-19 group (2.2% vs. 7.0%, *p* = 0.042). Survival status at last follow-up was comparable (74.5% vs. 75.6%, *p* = 0.795).

#### 3.2.5. Survival Comparison Between Pre-COVID-19 and COVID-19 Era Groups

**Figure 1:**
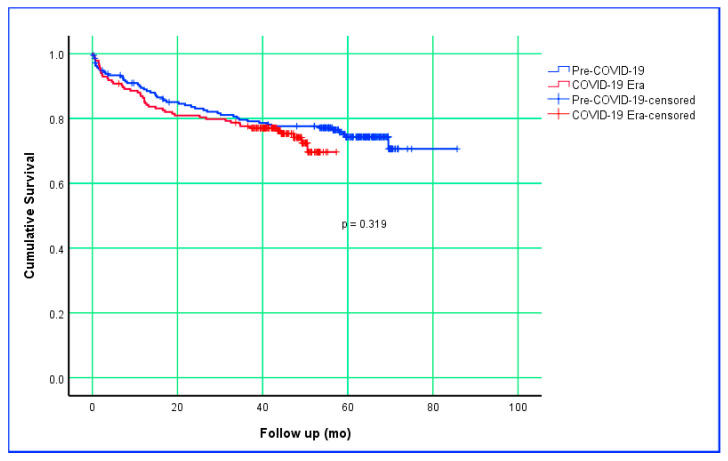
Kaplan–Meier survival curves comparing Pre–COVID-19 and COVID-19 Era groups.

Table 5 presents the overall survival analysis of patients who underwent CRC surgery in the Pre-COVID-19 and COVID-19 Era periods. The mean survival time was 68 months (95% CI: 64–73) in the Pre-COVID-19 group and 46 months (95% CI: 43–49) in the COVID-19 Era (*p* = 0.319). The 1-, 3-, and 5-year survival rates of patients in the Pre-COVID-19 period were 89.0%, 79.1%, and 74.3%, respectively. The 1-, 3-, and 4-year survival rates of patients in the COVID-19 period were 86.4%, 77.1%, and 72.4%, respectively; 5-year survival data for this group are not yet available. Survival analysis based on the Kaplan–Meier estimate is shown in Figure 1.

### 3.3. Survivors Vs. Non-Survivors Groups

#### 3.3.1. Demographic and Clinical Differences

Table 6 presents demographic and clinical differences between survivors and non-survivors. The median age was significantly higher in non-survivors (70 years, IQR: 64–76) compared to survivors (62 years, IQR: 55–69) (*p* < 0.001). Male patients were more prevalent in the non-surviving group (69.7%) than in the surviving group (55.7%, *p* = 0.014). Comorbidities were also more frequent among non-survivors, particularly pulmonary diseases (18.2% vs. 6.7%, *p* = 0.002) and cardiac conditions (26.3% vs. 13.4%, *p* = 0.005). ASA III and ASA IV scores were more frequent in the non-survivor group, whereas ASA I and ASA II scores were higher in the survivor group (*p* < 0.001).

#### 3.3.2. Tumor Characteristics and Stage

Table 6 and Table 7 also shows that non-survivors had a significantly higher proportion of advanced-stage tumors. Preoperative median CA 19-9 (*p* = 0.007) and CEA (*p* < 0.001) levels were higher in the non-survivor group. Similarly, the number of positive lymph nodes was also higher in the non-survivor group (*p* < 0.001). T4 tumors occurred more frequently in the non-surviving group (37.8% vs. 20.1%, *p* = 0.002). Lymph node (N2) involvement was markedly higher in non-survivors (46.9% vs. 13.9%, *p* < 0.001). Distant metastasis rates were also significantly greater (36.4% vs. 14.8%, *p* < 0.001). High-risk histopathological features, such as perineural invasion (45.8% vs. 34.0%, *p* = 0.037) and lymphovascular invasion (71.4% vs. 56.5%, *p* = 0.009), were more common among non-survivors.

#### 3.3.3. Surgical and Therapeutic Approaches

According to Table 7, emergency surgery was more frequently performed in non-survivors (38.4% vs. 19.5%, *p* < 0.001), indicating more critical clinical presentations. Open surgery was also more common in this group (67.7% vs. 46.3%, *p* = 0.001), whereas laparoscopic surgery was performed more often in survivors (50.0% vs. 28.3%). Adjuvant chemotherapy was significantly less frequent in non-survivors (54.3% vs. 73.4%, *p* = 0.001).

#### 3.3.4. Postoperative Outcomes and Complications

Table 7 shows that postoperative complications occurred more frequently in non-survivors (45.5% vs. 26.2%, *p* < 0.001). Notably, sepsis (11.1% vs. 3.4%, *p* = 0.006) and metabolic complications (10.1% vs. 1.3%, *p* < 0.001) were significantly increased. Hospital stay was longer in the non-survivor group (*p* = 0.010), whereas the total follow-up duration was, as expected, higher in the survivor group (*p* < 0.001).

#### 3.3.5. Factors Influencing Mortality in Patients with CRC

Table 8 presents logistic regression analysis, identifying multiple factors associated with increased odds of mortality. Distant metastasis emerged as a significant factor (*p* = 0.008, OR = 2.86, 95% CI: 1.32–6.16). Poorly differentiated (*p* = 0.023, OR = 4.57, 95% CI: 1.24–16.83) and undifferentiated tumors (*p* = 0.028, OR = 6.95, 95% CI: 1.24–39.05) were strongly linked to higher mortality odds. Lymph node involvement was also associated with increased mortality (*p* = 0.001, OR = 1.14, 95% CI: 1.06–1.22), along with intestinal perforation (*p* < 0.001, OR = 11.76, 95% CI: 3.76–36.79) and bowel obstruction (*p* = 0.007, OR = 2.67, 95% CI: 1.30–5.47). The Hosmer–Lemeshow test (Chi-square: 9.30, *p* = 0.315) confirmed adequate model calibration. Overall, these findings underscore the prognostic importance of advanced tumor stage, metastasis, urgent surgical conditions, and systemic disease burden in CRC mortality, reinforcing the need for early detection and timely intervention in high-risk patients.

### 3.4. Metastatic Vs. Non-Metastatic Groups

#### 3.4.1. Demographic and Clinical Features

Table 9 presents a comparison of quantitative variables between metastatic and non-metastatic CRC patients. Non-metastatic group were significantly older (median 64 years, 95% CI: 63–66) compared to metastatic group (59 years, 95% CI: 54–65, *p* = 0.001). Median hospital stay was also longer for metastatic patients (12 days, 95% CI: 10–15) versus non-metastatic patients (8 days, 95% CI: 8–10, *p* < 0.001). Median follow-up duration was shorter for metastatic group (44 months, 95% CI: 34–50) compared to non-metastatic group (50 months, 95% CI: 48–53, *p* = 0.002).

#### 3.4.2. Tumor Characteristics and Stage

Table 9 and Table 10 collectively demonstrate distinct tumor-related differences between metastatic and non-metastatic CRC patients. Preoperative median CEA levels were markedly elevated in metastatic cases (6 ng/mL, 95% CI: 4–16) compared to non-metastatic cases (2 ng/mL, 95% CI: 2–3, *p* < 0.001). Tumor size was generally larger in the metastatic group (50 mm, 95% CI: 47–56) versus the non-metastatic group (45 mm, 95% CI: 45–50, *p* = 0.054). The median number of positive LN retrieved was substantially higher in metastatic patients (3, 95% CI: 2–5) than in non-metastatic patients (0, 95% CI: 0–1, *p* < 0.001). Moreover, advanced T4 stage tumors were significantly more prevalent among metastatic patients (65.8% vs. 14.1%, *p* < 0.001), with lymph node (N2) involvement (47.5% vs. 15.7%, *p* < 0.001). Poor tumor differentiation was also more frequent in the metastatic group (22.4% vs. 10.0%, *p* = 0.001). Adverse pathological features such as perineural invasion (67.5% vs. 29.4%, *p* < 0.001) and lymphovascular invasion (89.9% vs. 52.7%, *p* < 0.001) were also notably more common in the metastatic group.

#### 3.4.3. Surgical and Therapeutic Approaches

Open surgery was performed more frequently in metastatic patients (81.3%) compared to non-metastatic patients (44.2%, *p* < 0.001). Conversely, laparoscopic surgery was more common in the non-metastatic group (52.4% vs. 13.8%). Rates of emergency surgery, intestinal obstruction, and perforation did not differ significantly between groups.

#### 3.4.4. Postoperative Outcomes and Complications

Metastatic patients exhibited higher rates of overall postoperative complications (52.5% vs. 25.6%, *p* < 0.001), wound infection (22.5% vs. 10.4%, *p* = 0.007), and sepsis (10.0% vs. 4.1%, *p* = 0.048). While the hospital stay was significantly longer in the metastatic group (*p* < 0.001), the postoperative follow-up duration was longer in the non-metastatic group (*p* = 0.002).

#### 3.4.5. Factors Influencing Metastasis in Patients with CRC

Table 11 summarizes the multivariate analysis, which identified preoperative CEA level (*p* = 0.002, OR = 1.02, 95% CI: 1.01–1.05), lymph node involvement (*p* = 0.002, OR = 4.87, 95% CI: 1.83–12.95), and perineural invasion (*p* = 0.033, OR = 2.17, 95% CI: 1.07–4.45) as factors associated with increased odds of metastasis. The Hosmer and Lemeshow test indicated good model fit (Chi-square: 4.47, *p* = 0.812).

## 4. Discussion

Although a limited number of opposing views exist, the prevailing consensus in the literature is that the COVID-19 pandemic significantly impacted the diagnosis, treatment strategies, postoperative complications, recurrence, and survival outcomes of CRC [34,43]. The suspension of screening programs, delays in diagnostic processes, and changes in treatment options contributed to an increased disease burden by leading more patients to present with advanced-stage CRC [34]. Restrictions in colonoscopy access further delayed diagnoses, resulting in more aggressive tumor behavior and higher rates of late-stage disease [44]. Additionally, patients’ reluctance to seek medical care due to concerns about infection exacerbated diagnostic delays, leading to more frequent presentations with complications requiring emergency surgical intervention [29,44]. In the present multicentric study, although colonoscopic screening metrics were not directly measured, the absence of a decline in the number of CRC surgeries performed during the same pre- and post-pandemic periods suggests that screening programs in the three participating centers remained stable. Furthermore, the lack of significant delay between diagnosis and surgery (*p* = 0.526) and from surgery to pathology reporting (*p* = 0.085) indicates that treatment continuity was maintained, in line with reports in the literature [45]. However, during the COVID-19 era, tumors were significantly larger (*p* < 0.001), with higher rates of lymph node (N2) involvement (*p* = 0.027), poor differentiation (*p* = 0.030) and perineural invasion (*p* = 0.001). These findings suggest that, beyond healthcare access issues, biological and tumor-related factors—possibly influenced by genetic or environmental conditions—may have contributed to the higher stage at presentation during the pandemic. This may also reflect the influence of other unexamined epidemiological and epigenetic factors. Confirming these hypotheses will require large-scale, population-based, multicenter studies with standardized data collection and long-term follow-up.

Following the WHO’s declaration of the COVID-19 pandemic, changes occurred in surgical practice as in all areas of healthcare management. In the early stages of the pandemic, global concerns regarding aerosolization risk, limitations in personal protective equipment, and restricted intensive care unit capacity led to hesitancy toward minimally invasive techniques, resulting in a decline in laparoscopic CRC surgery rates [28,33,34,46]. However, as the pandemic progressed, studies emerged demonstrating that laparoscopic surgery could be performed safely with appropriate protective measures. These reports highlighted advantages such as shorter hospital stays and reduced risk of postoperative pulmonary complications. In the present study, a decline in laparoscopic surgery rates (48.8% vs. 39.7%) and an increase in open surgery rates (47.4% vs. 56.5%) were observed during the pandemic; however, these changes did not reach statistical significance (*p* = 0.179). These findings suggest that our centers maintained the use of minimally invasive approaches for selected cases under standardized protective measures, while resource constraints contributed to a nuanced shift in surgical approach selection.

During the pandemic, changes occurred in the timing and prioritization of CRC surgery. In the early phase, patients’ reluctance to visit hospitals and the postponement of some elective cancer surgeries led to an increase in general CRC-related complications such as obstruction and perforation, with studies reporting a higher need for emergency surgery due to tumor-related presentations [43,46]. Conversely, other reports demonstrated that with early diagnosis and appropriate triage protocols, the need for emergency surgery did not increase, and in some cases, including our study, even decreased [24,34]. Similarly, findings regarding ostomy formation during the pandemic have been inconsistent. Some studies suggested a higher frequency of ostomy creation to protect anastomoses and reduce hospital stays—findings that were sometimes statistically significant—while others reported unchanged or even reduced ostomy rates in the early stages [23,28,33,34,43,44,46,47,48,49]. In the present study, although not statistically significant, emergency surgery rates decreased from 26.3% before the pandemic to 21.7% during COVID-19 (*p* = 0.291). Intestinal obstruction rates remained similar between periods (*p* = 0.937), but notably, perforation rates significantly declined during the pandemic (*p* = 0.042), consistent with some previous reports [24,35]. This reduction in perforation, despite larger tumor sizes, may be attributed to rapid responses to emergency admissions, prompt diagnosis-to-surgery management, and effective in-hospital alert and consultation systems. Additionally, the tendency for patients to present directly to emergency departments rather than outpatient clinics during the pandemic may have facilitated surgical intervention before perforation developed.

Significant research has been published on how declines in cancer screening, changes in treatment approaches, delays in therapy, and adjustments in follow-up protocols during the pandemic affected tumor staging and tumor biology [43]. In the context of CRC, numerous studies have shown that the pandemic led to decreased colonoscopic screening, detection of larger and more advanced tumors, changes in clinical presentation, and temporary shifts from surgical to neoadjuvant therapy [28,50]. However, some reports suggest that after the first pandemic wave, these disruptions stabilized, with less impact than initially expected. In contrast to many previous studies, the present multicentric analysis—though not reaching statistical significance—found a proportional decrease in the use of neoadjuvant therapy during the pandemic. Furthermore, increases were observed in tumor size, lymph node involvement, poor differentiation, and markers of tumor aggressiveness such as perineural invasion. Nevertheless, this study did not demonstrate an effect of the pandemic on distant metastases, overall survival, or disease-specific survival, though longer follow-up is needed for metastasis and survival assessment. Considering the doubling time of CRC, which ranges from 112 to 404 days (median: 211 days), it is unlikely that the biological behavior of the tumor would change dramatically within a short period [51]. Since our study covers the first 18 months of the pandemic—approximately the doubling time period—it offers preliminary insights into its impact on tumor biology. However, stronger conclusions will require both long-term results of this study and post-pandemic epidemiological data. To conclude with a key question: given that many cancer studies in the literature report indirect effects of the pandemic on tumor characteristics, can this truly be considered coincidental?

Regardless of the pandemic, the most common postoperative complications following CRC surgery include intestinal obstruction, anastomotic leakage, intra-abdominal abscess, pulmonary complications, wound infection, sepsis, and metabolic complications [52,53]. There are limited studies examining how these complications changed during the pandemic period. Uyan et al. [33] reported higher postoperative complication rates during the early pandemic (*p* = 0.014) nd similar findings were observed by Ergün et al. [43] (*p* = 0.015). In contrast, Ferahman et al. [44] found no significant difference in overall complication rates. In the present study, the overall complication rate was not significantly affected by the pandemic period (*p* = 0.385). No significant differences were observed between periods for specific complications, including intestinal obstruction, anastomotic leakage, intra-abdominal abscess, pulmonary complications, wound infection, sepsis, and metabolic complications (only metabolic complications approached significance; *p* = 0.055). Ostomy decisions were individualized based on tumor location, patient physiological reserve, and the surgical risk–benefit balance; ostomy rates did not differ significantly between periods (*p* = 0.243). This consistency suggests that, even under crisis conditions, decisions regarding anastomosis versus ostomy could be maintained in line with oncologic and technical principles.

Numerous studies have examined whether the COVID-19 pandemic influenced postoperative survival and mortality rates in CRC surgery, with most reporting no significant differences, largely attributed to short follow-up periods [46,50]. However, some studies have reported increased mortality during the pandemic period [28,44]. In the survival analyses of the present study, no statistically significant difference was found between the pandemic and pre-pandemic periods in terms of mean survival times (*p* = 0.319). Similarly, mortality rates did not differ between periods (*p* = 0.795). This finding suggests that maintaining the timeline across the care pathway—from diagnosis to surgery and pathology reporting—may offset short- to mid-term oncologic outcomes, even in the presence of more advanced stages and larger tumors. Nevertheless, the relatively shorter median follow-up time in the post-pandemic group (*p* < 0.001) warrants caution, as some late effects may not yet be fully captured. Therefore, long-term follow-up data from this study should be awaited for more definitive conclusions. 

Independent of the COVID-19 pandemic, this multicentric, high-volume study places significant importance on examining factors associated with mortality and metastasis in CRC and assessing whether these outcomes were influenced by the pandemic, with the pandemic period considered as an independent variable. Factors related to mortality in CRC include age, tumor location, tumor stage, and the need for emergency surgical intervention. Multivariate logistic regression analysis clearly identified independent predictors of mortality: each increase in the number of positive LN retrieved was found to increase the odds of mortality (OR = 1.14); worsening tumor differentiation was associated with a stepwise elevation in odds (moderate differentiation OR = 2.99, poor differentiation OR = 4.57, undifferentiated OR = 6.95); preoperative intestinal obstruction (OR = 2.67) and perforation (OR = 11.76) markedly increased the odds; and distant metastasis was linked to higher odds of mortality (OR = 2.86). In a separate multivariate analysis for factors associated with metastasis development, higher preoperative blood CEA level (OR = 1.02), lymph node involvement (OR = 4.87), and perineural invasion (OR = 2.17) were associated with increased odds of metastasis, supporting the close association between biomarkers of biological aggressiveness and systemic dissemination of the cancer.

Screening and diagnosis of CRC are essential for early detection and optimal treatment. However, conventional modalities—including digital rectal examination, colonoscopy, fecal-based tests [fecal immunochemical test (FIT) and guaiac-based fecal occult blood test (gFOBT)], Colon capsule endoscopy, CT colonography, CT, MRI, and histopathology—each have variable sensitivity, specificity, and reproducibility, and many are operator-dependent, leading to the absence of a universally accepted gold standard approach [54,55,56,57,58,59]. Although not widely used for screening and diagnosis during the pandemic, in recent years artificial intelligence (AI)—particularly machine learning (ML) and deep learning (DL)—has been increasingly applied to a broad range of benign and malignant diseases to enhance risk prediction, automate image interpretation, and support clinical decision-making [59]. Several recent studies have demonstrated the value of AI systems in radiology, pathology, endoscopy-colonoscopy, and surgical planning, laying the groundwork for precision medicine [60,61,62,63,64]. In CRC specifically, DL-based algorithms have been applied across multiple domains [65]. In radiology, they are used for CT- and MRI-based automated tumor segmentation, nodal staging, and treatment response prediction. In pathology, they enable slide-level classification and prediction of molecular biomarkers such as microsatellite instability (MSI) [66]. In digital pathology, many studies report clinically relevant sensitivity and specificity for DL models, with several commercially available tools nearing clinical adoption [66,67,68]. In endoscopy, real-time computer-aided detection (CADe) systems have consistently improved adenoma detection rates in trials, and at least one (GI Genius) has received FDA approval [69,70,71,72]. Together, these advances suggest that integrating DL with standardized imaging and pathology workflows may further enhance early detection and streamline therapeutic decision-making; nevertheless, external validation, domain adaptation, and prospective multicenter trials remain crucial before widespread implementation.

Recent literature has begun to explore, beyond the well-established CRP and PCT markers, the role of newer biomarkers such as presepsin [73] and butyrylcholinesterase as prognostic indicators in colorectal surgery for postoperative infectious complications [74]. For example, a 2024 prospective single-center study by Verras et al. [75] found that lower butyrylcholinesterase levels on the first and third days after colorectal surgery were significantly associated with heightened risk of surgical site infection and severity of the infection. In another study, Alburiahi et al. [76] reported that reduced preoperative cholinesterase levels correlated with poorer prognosis in CRC patients. These findings suggest potential for butyrylcholinesterase to act as an early warning marker, enabling more proactive postoperative monitoring or targeted prophylactic interventions. However, in our multicentric cohort, butyrylcholinesterase levels were not measured, so we could not include this parameter in our analyses. Future prospective studies in our centers will be needed to collect perioperative butyrylcholinesterase data, validate its clinical utility, and potentially integrate it into risk stratification tools for postoperative care.

Overall, our study demonstrates that during the pandemic period there was an increased tendency for patients to present with larger and more advanced-stage tumors, as well as a higher frequency of certain aggressive histopathological features (such as perineural invasion and poor differentiation). In contrast, surgical approaches and perioperative outcomes remained stable, largely due to institutional prioritization and effective process management. This balance underscores the importance of multidisciplinary coordination, flexible resource planning, and standardized emergency care pathways for sustaining oncological care under crisis conditions. In systemic shocks similar to a pandemic, rapidly reactivating screening capacity, updating prioritization algorithms based on data, and establishing seamless diagnosis–treatment bridges for high-risk groups will be critical in limiting both stage migration and the worsening of biological aggressiveness patterns. In this context, the quantitative indicators obtained from our series (e.g., increased tumor size, higher rates of T4 and N2 stages, increased perineural invasion, stable complication profile, and unchanged mortality/survival rates) concretely reflect the resilience and adaptability of our institutions.

### 4.1. Limitations

Despite the strengths of this study, certain limitations should be acknowledged. First, as a retrospective study, our findings are inherently subject to selection bias and limitations in data accessibility. Second, variations in inter-institutional treatment protocols during the pandemic may have influenced patient outcomes, making direct comparisons between centers more challenging. Third, the study primarily focuses on short- and mid-term outcomes; longer follow-up is necessary to fully evaluate the pandemic’s impact on disease recurrence and overall survival. Fourth, because complete postoperative mid-term data from all three participating centers were not fully available, precise information on local recurrence could not be obtained. In addition, there were insufficient data on the occurrence of distant organ metastases after surgery during the pandemic period; thus, patients could not be compared with respect to this outcome. Fifth, due to the retrospective study design, despite the absence of major differences between groups in many aspects, the exact reasons for the more advanced disease stage observed in patients during the pandemic could not be fully elucidated. Finally, although a comprehensive set of clinical and pathological parameters was included, potential confounding variables such as socioeconomic factors and healthcare accessibility were not explicitly analyzed; these factors could provide better context for the differences observed during the pandemic.

### 4.2. Lessons Learned from the Pandemic and Future Directions

CRC, one of the most prevalent malignancies worldwide, and the COVID-19 pandemic, responsible for millions of deaths, represent two distinct yet interrelated clinical challenges. The pandemic significantly disrupted the management of CRC patients at every stage, from screening to treatment strategies [77]. Public health measures such as stay-at-home orders, social distancing, and personal protective equipment were critical for immunosuppressed patients undergoing CRC or other cancer treatments [2]. However, the postponement of CRC screening to redirect healthcare resources and minimize infection risks led to delayed diagnoses and treatment initiation, posing a major problem [57].

To better prepare for future pandemics and minimize their impact on the management of CRC and other cancers, proactive health policies and strategies are essential [78]. In the context of CRC, policies should focus on raising public awareness of symptoms and risk factors, expanding the use of non-invasive screening tools such as FIT, integrating telemedicine into routine patient care, and establishing pandemic-resilient healthcare facilities [79,80,81]. Enhancing home-based patient monitoring and optimizing multidisciplinary collaboration through teleconferencing are also key steps to ensure continuity of CRC care.

Although CRC cases underwent surgery during the COVID-19 Era presented with more advanced tumor characteristics, the absence of differences between groups in key epidemiological features and overall survival remains a puzzling finding and should be interpreted in the context of long-term follow-up data and future multicenter studies.

AI-based algorithms represent a key component of future pandemic-resilient CRC care. These tools can not only identify high-risk individuals and facilitate early diagnosis [82,83] but also support triage, predict treatment response, and optimize resource allocation in times of healthcare strain. Future efforts should prioritize the integration of DL models for transrectal ultrasound, CT and MRI-based staging, digital pathology for MSI prediction, and CADe-assisted colonoscopy into routine practice. Such integration could help maintain screening performance and treatment timelines even during crises [84,85,86]. Large, multicenter, prospective studies and robust external validation are essential to confirm generalizability and ensure safe, equitable deployment before widespread clinical adoption.

## 5. Conclusions

This multicenter study demonstrates that surgical timing remained stable during the COVID-19 Era; however, patients who underwent surgery in the COVID-19 Era presented with more advanced pathology—higher rates of T4 tumors, more N2 nodal involvement, more frequent perineural invasion, poorer differentiation, and larger primary tumors. In multivariable analyses, poor differentiation, distant metastasis, intestinal obstruction, and intestinal perforation were independent predictors of mortality, whereas elevated preoperative CEA, lymph node involvement, and perineural invasion independently predicted metastasis. Preoperative intestinal perforation was less frequent in the COVID-19 Era group. These findings highlight that, although treatment timing and survival outcomes did not differ between groups, patients during the pandemic period exhibited more aggressive tumor biology. This underlines the importance of continued surveillance, early diagnosis, and risk stratification strategies to prevent adverse oncological outcomes in future healthcare crises.

## Figures and Tables

**Table 1:** Continuous demographic and clinical characteristics of the study population.

Parameters [Median (95%CI)]	Median	IQR	95% CI
Age	64	53–73	63–66
From diagnosis to surgery (days)	8	5–14	8–10
From surgery to pathology (days)	22	14–34	21–25
Preoperative CA19.9	22.5	8–125	18–34
Preoperative CEA	2	1–7	2–3
Tumor size (mm)	45	33–60	45–50
Total LN retrieved (number)	23	14–38	22–26
Positive LN retrieved (number)	1	1–3	1–2
Hospital stay (days)	9	7–13	9–10
Follow up (months)	49	38–59	47–51

CA 19-9: Carbohydrate Antigen 19-9, CEA: Carcinoembryonic Antigen, CI: Confidence Interval, IQR: Interquartile Range, LN: Lymph node.

**Table 2:** Categorical demographic and clinical characteristics of the study population.

Variables	Categories	Number (%)
Groups	Pre-COVID-19	213 (53.7)
	COVID-19 Era	184 (46.4)
Center	Inonu University	210 (52.9)
	Elazig City Hospital	69 (17.4)
	Firat University	118 (29.7)
Gender	Male	235 (59.2)
	Female	162 (40.8)
Overall comorbidity	Absent	207 (52.1)
	Present	190 (47.9)
DM	Absent	317 (79.9)
	Present	80 (20.2)
HT	Absent	295 (74.3)
	Present	102 (25.7)
Pulmonary	Absent	359 (90.4)
	Present	38 (9.6)
Cardiac	Absent	331 (83.4)
	Present	66 (16.6)
Thyroid	Absent	365 (91.9)
	Present	32 (8.1)
ASA Score	ASA I	20 (5.0)
	ASA II	167 (42.1)
	ASA III	203 (51.1)
	ASA IV	7 (1.8)
Tumor locations	Transverse Colon	6 (1.5)
	Sigmoid	79 (19.9)
	Right Colon	85 (21.4)
	Rectum	137 (34.5)
	Rectosigmoid	18 (4.5)
	Left Colon	32 (8.1)
	Cecum	40 (10.1)
Timing of surgery	Elective	301 (75.8)
	Emergency	96 (24.2)
Intestinal obstruction (preop)	Absent	320 (80.6)
	Present	77 (19.4)
Intestinal perforation (preop)	Absent	378 (95.2)
	Present	19 (4.8)
Type of surgery	Open	205 (51.6)
	Laparoscopic	177 (44.6)
	Conversion	15 (3.8)
Ostomy (during index surgery)	Absent	203 (51.1)
	Present	194 (48.9)
Mucinous component	Absent	337 (87.1)
	Present	50 (12.9)
Tumor differentiation	Well	81 (21.0)
	Moderately	247 (64.0)
	Poor	48 (12.4)
	Undifferentiated	10 (2.6)
Primary tumor (T)	T1	15 (3.8)
	T2	47 (12.0)
	T3	230 (58.7)
	T4	96 (24.5)
	Tis	4 (1.0)
Lymph node involvement (N)	N0	195 (49.6)
	N1	110 (28.0)
	N2	87 (22.1)
	N3	1 (0.3)
Distant metastasis (M)	M0	317 (79.9)
	M1	80 (20.2)
Perineural Invasion	Absent	246 (63.1)
	Present	144 (36.9)
Lymphovascular invasion	Absent	156 (39.8)
	Present	236 (60.2)
Overall complications (postop)	Absent	274 (69.0)
	Present	123 (31.0)
Intestinal obstruction (postop)	Absent	375 (94.5)
	Present	22 (5.5)
Anastomotic leak (postop)	Absent	385 (97.0)
	Present	12 (3.0)
Intraabdominal abscess (postop)	Absent	379 (95.5)
	Present	18 (4.5)
Pulmonary complications (postop)	Absent	375 (94.5)
	Present	22 (5.5)
Wound infection (postop)	Absent	346 (87.2)
	Present	51 (12.9)
Sepsis (postop)	Absent	376 (94.7)
	Present	21 (5.3)
Metabolic complications (postop)	Absent	383 (96.5)
	Present	14 (3.5)
Neoadjuvant therapy	Absent	320 (82.9)
	Present	66 (17.1)
Adjuvant CT	Absent	117 (31.5)
	Present	255 (68.6)
Adjuvant RT	Absent	332 (89.3)
	Present	40 (10.8)
Outcomes	Alive	298 (75.1)
	Dead	99 (24.9)

ASA: American Society of Anesthesiologists, CT: Chemotherapy, DM: Diabetes Mellitus, HT: Hypertension, Preop: Preoperative, Postop: Postoperative, RT: Radiotherapy. The percentages were calculated from the subset of patients with complete data, since information for some cases was unavailable.

**Table 3:** Comparison of continuous variables between Pre-COVID-19 and COVID-19 era groups.

Parameters [Median (95%CI)]	Pre-COVID-19	COVID-19 Era	*p* *
Age	63 (62–65)	65 (63–69)	0.579
From diagnosis to surgery (days)	8 (8–10)	8 (8–10)	0.526
From surgery to pathology (days)	21 (18–25)	22 (20–27)	0.085
Preoperative CA19-9	22 (17–38)	25 (17–39)	0.758
Preoperative CEA	2 (2–3)	3 (3–5)	0.128
Tumor size (mm)	42 (40–45)	50 (50–58)	<0.001
Total LN retrieved (number)	21 (19–24)	26 (23–29)	0.006
Positive LN retrieved (number)	0 (0–0)	1 (1–2)	0.115
Hospital stay (days)	9 (8–10)	8 (8–10)	0.422
Follow up (months)	58 (57–60)	44 (42–44)	<0.001

CA 19-9: Carbohydrate Antigen 19-9, CEA: Carcinoembryonic Antigen, CI: Confidence Interval, IQR: Interquartile Range, LN: Lymph node, * Mann–Whitney U test.

**Table 4:** Comparison of categorical variables between Pre-COVID-19 and COVID-19 era groups.

Parameters	Categories (n; %)	Pre-COVID-19	COVID-19 Era	*p*
Center	Inonu University	102 (47.9)	108 (58.7)	0.064 *
	Elazig City Hospital	44 (20.7)	25 (13.6)	
	Firat University	67 (31.5)	51 (27.7)	
Gender	Male	128 (60.1)	107 (58.2)	0.695 *
	Female	85 (39.9)	77 (41.9)	
Overall comorbidity	Absent	106 (49.8)	101 (54.9)	0.308 *
	Present	107 (50.2)	83 (45.1)	
DM	Absent	172 (80.8)	145 (78.8)	0.630 *
	Present	41 (19.3)	39 (21.2)	
HT	Absent	156 (73.2)	139 (75.5)	0.600 *
	Present	57 (26.8)	45 (24.5)	
Pulmonary	Absent	193 (90.6)	166 (90.2)	0.999 **
	Present	20 (9.4)	18 (9.8)	
Cardiac	Absent	180 (84.5)	151 (82.1)	0.515 *
	Present	33 (15.5)	33 (17.9)	
Thyroid	Absent	194 (91.1)	171 (92.9)	0.623 **
	Present	19 (8.9)	13 (7.1)	
ASA Score	ASA I	10 (4.7)	10 (5.4)	0.579 *
	ASA II	90 (42.3)	77 (41.9)	
	ASA III	111 (52.1)	92 (50.0)	
	ASA IV	2 (0.9)	5 (2.7)	
Tumor locations	Transverse Colon	4 (1.9)	2 (1.1)	0.724 *
	Sigmoid	42 (19.7)	37 (20.1)	
	Right Colon	44 (20.7)	41 (22.3)	
	Rectum	78 (36.6)	59 (32.07)	
	Rectosigmoid	9 (4.2)	9 (4.9)	
	Left Colon	19 (8.9)	13 (7.1)	
	Cecum	17 (8.0)	23 (12.5)	
Timing of surgery	Elective	157 (73.7)	144 (78.3)	0.291 *
	Emergency	56 (26.3)	40 (21.7)	
Intestinal obstruction (preop)	Absent	172 (80.8)	148 (80.4)	0.937 *
	Present	41 (19.3)	36 (19.6)	
Intestinal perforation (preop)	Absent	198 (93.0)	180 (97.8)	0.042 **
	Present	15 (7.0)	4 (2.2)	
Type of surgery	Open	101 (47.4)	104 (56.5)	0.179 *
	Laparoscopic	104 (48.8)	73 (39.7)	
	Conversion	8 (3.8)	7 (3.8)	
Ostomy (during index surgery)	Absent	103 (48.4)	100 (54.4)	0.243 *
	Present	110 (51.6)	84 (45.7)	
Mucinous component	Absent	188 (90.0)	149 (83.7)	0.068 *
	Present	21 (10.1)	29 (16.3)	
Tumor differentiation	Well	44 (21.15)	37 (20.8)	0.030 *
	Moderately	143 (68.8)	104 (58.4)	
	Poor	17 (8.17)	31 (17.4)	
	Undifferentiated	4 (1.9)	6 (3.4)	
Primary tumor (T)	T1	12 (5.7)	3 (1.7)	0.007 *
	T2	29 (13.8)	18 (9.9)	
	T3	128 (61.0)	102 (56.0)	
	T4	38 (18.1)	58 (31.9)	
	Tis	3 (1.4)	1 (0.6)	
Lymph node involvement (N)	N0	108 (51.4)	87 (47.5)	0.027 *
	N1	66 (31.4)	44 (24.0)	
	N2	35 (16.7)	52 (28.4)	
	N3	1 (0.5)	0 (0.0)	
Distant metastasis (M)	M0	176 (82.6)	141 (76.6)	0.137 *
	M1	37 (17.4)	43 (23.4)	
Perineural Invasion	Absent	147 (70.3)	99 (54.7)	0.001 *
	Present	62 (29.7)	82 (45.3)	
Lymphovascular invasion	Absent	88 (41.9)	68 (37.4)	0.360 *
	Present	122 (58.1)	114 (62.6)	
Overall complications (postop)	Absent	151 (70.9)	123 (66.9)	0.385 *
	Present	62 (29.1)	61 (33.15)	
Intestinal obstruction (postop)	Absent	204 (95.8)	171 (92.9)	0.311 **
	Present	9 (4.2)	13 (7.1)	
Anastomotic leak (postop)	Absent	208 (97.7)	177 (96.2)	0.581 **
	Present	5 (2.4)	7 (3.8)	
Intraabdominal abscess (postop)	Absent	202 (94.8)	177 (96.2)	0.684 **
	Present	11 (5.2)	7 (3.8)	
Pulmonary complications (postop)	Absent	200 (93.9)	175 (95.1)	0.759 **
	Present	13 (6.1)	9 (4.9)	
Wound infection (postop)	Absent	180 (84.5)	166 (90.2)	0.122 **
	Present	33 (15.5)	18 (9.8)	
Sepsis (postop)	Absent	201 (94.4)	175 (95.1)	0.917 **
	Present	12 (5.6)	9 (4.9)	
Metabolic complications (postop)	Absent	209 (98.1)	174 (94.6)	0.055 **
	Present	4 (1.9)	10 (5.4)	
Neoadjuvant therapy	Absent	163 (79.5)	157 (86.7)	0.081 **
	Present	42 (20.5)	24 (13.3)	
Adjuvant CT	Absent	60 (30.5)	57 (32.6)	0.661 *
	Present	137 (69.5)	118 (67.4)	
Adjuvant RT	Absent	177 (89.9)	155 (88.6)	0.692 *
	Present	20 (10.2)	20 (11.4)	
Outcomes	Alive	161 (75.6)	137 (74.5)	0.795 *
	Dead	52 (24.4)	47 (25.5)	

ASA: American Society of Anesthesiologists, CT: Chemotherapy, DM: Diabetes Mellitus, HT: Hypertension, Preop: Preoperative, Postop: Postoperative, RT: Radiotherapy, *: Pearson chi square test; **: Chi-square test with Yates correction.

**Table 5:** Mean survival time with standard errors and 95% confidence intervals for Pre-COVID-19 and COVID-19 era groups.

Groups	Mean (Months)				*p*
	Estimate	Std. Error	95% CI		
			Lower Bound	Upper Bound	
Pre-COVID-19	68	2.2	64	73	0.319
COVID-19 Era	46	1.5	43	49	
Overall	67	1.7	64	70	

**Table 6:** Comparison of continuous variables between survivor and non-survivor subgroups.

Parameters [Median (95%CI)]	Survivor	Non-Survivor	*p* *
From diagnosis to surgery (days)	8 (8–10)	8(7–10)	0.873
From surgery to pathology (days)	22 (20–25)	20 (18–24)	0.184
Age (years)	62 (61–64)	70 (67–74)	<0.001
Preoperative CA19.9	21 (17–31)	67.5 (20–121)	0.007
Preoperative CEA	2 (2–3)	4 (3–8)	<0.001
Tumor size (mm)	45 (45–50)	50 (50–56)	0.092
Total LN retrieved (number)	24 (22–28)	23 (21–27)	0.288
Positive LN retrieved (number)	0 (0–0)	3 (2–5)	<0.001
Hospital stay (days)	8 (8–10)	10 (9–13)	0.010
Follow up (months)	54 (52–55)	12 (11–17)	<0.001

CA 19-9: Carbohydrate Antigen 19-9, CEA: Carcinoembryonic Antigen, CI: Confidence Interval, IQR: Interquartile Range, LN: Lymph node, * Mann–Whitney U test.

**Table 7:** Comparison of categorical variables between survivor and non-survivor subgroups.

Parameters	Categories	Survivor	Non-Survivor	*p*
Groups	Pre-COVID-19	161 (54.0)	52 (52.5)	0.795 *
	COVID-19 Era	137 (46.0)	47 (47.5)	
Gender	Male	166 (55.7)	69 (69.7)	0.014 *
	Female	132 (44.3)	30 (30.3)	
Overall comorbidity	Absent	163 (54.7)	44 (44.4)	0.077 *
	Present	135 (45.3)	55 (55.6)	
DM	Absent	238 (79.9)	79 (79.8)	0.999 **
	Present	60 (20.1)	20 (20.2)	
HT	Absent	227 (76.2)	68 (68.7)	0.140 *
	Present	71 (23.8)	31 (31.3)	
Pulmonary	Absent	278 (93.3)	81 (81.8)	0.002 **
	Present	20 (6.7)	18 (18.2)	
Cardiac	Absent	258 (86.6)	73 (73.7)	0.005 **
	Present	40 (13.4)	26 (26.3)	
Thyroid	Absent	273 (91.6)	92 (92.9)	0.838 **
	Present	25 (8.4)	7 (7.1)	
ASA	ASA I	18 (6.0)	2 (2.0)	<0.001 *
	ASA II	141 (47.3)	26 (26.3)	
	ASA III	135 (45.3)	68 (68.7)	
	ASA IV	4 (1.3)	3 (3.0)	
Tumor locations	Transverse Colon	4 (1.3)	2 (2.0)	0.083 *
	Sigmoid	64 (21.5)	15 (15.2)	
	Right Colon	60 (20.1)	25 (25.3)	
	Rectum	110 (36.9)	27 (27.3)	
	Rectosigmoid	11 (3.7)	7 (7.1)	
	Left Colon	23 (7.72)	9 (9.1)	
	Cecum	26 (8.72)	14 (14.1)	
Timing of surgery	Elective	240 (80.5)	61 (61.6)	<0.001 *
	Emergency	58 (19.5)	38 (38.4)	
Intestinal obstruction (preop)	Absent	249 (83.6)	71 (71.7)	0.015 **
	Present	49 (16.4)	28 (28.3)	
Intestinal perforation (preop)	Absent	289 (97.0)	89 (89.9)	0.010 **
	Present	9 (3.02)	10 (10.1)	
Type of surgery	Open	138 (46.3)	67 (67.7)	0.001
	Laparoscopic	149 (50.0)	28 (28.3)	
	Conversion	11 (3.7)	4 (4.0)	
Ostomy (during index surgery)	Absent	156 (52.4)	47 (47.5)	0.401 *
	Present	142 (47.7)	52 (52.5)	
Mucinous component	Absent	258 (89.0)	79 (81.4)	0.056 *
	Present	32 (11.0)	18 (18.6)	
Tumor differentiation	Well	75 (25.9)	6 (6.3)	<0.001 *
	Moderately	182 (62.8)	65 (67.7)	
	Poor	26 (9.0)	22 (22.9)	
	Undifferentiated	7 (2.4)	3 (3.1)	
Primary tumor (T)	T1	14 (4.8)	1 (1.0)	0.002 *
	T2	40 (13.61)	7 (7.1)	
	T3	177 (60.2)	53 (54.1)	
	T4	59 (20.1)	37 (37.8)	
	Tis	4 (1.4)	0 (0.0)	
Lymph node involvement (N)	N0	165 (55.9)	30 (30.6)	<0.001 *
	N1	88 (29.8)	22 (22.5)	
	N2	41 (13.9)	46 (46.9)	
	N3	1 (0.3)	0 (0.0)	
Distant metastasis (M)	M0	254 (85.2)	63 (63.6)	<0.001 *
	M1	44 (14.8)	36 (36.4)	
Perineural invasion	Absent	194 (66.0)	52 (54.2)	0.037 *
	Present	100 (34.0)	44 (45.8)	
Lymphovascular invasion	Absent	128 (43.5)	28 (28.6)	0.009 *
	Present	166 (56.5)	70 (71.4)	
Overall complications (postop)	Absent	220 (73.8)	54 (54.6)	<0.001 *
	Present	78 (26.2)	45 (45.5)	
Intestinal obstruction (postop)	Absent	280 (94.0)	95 (96.0)	0.617 **
	Present	18 (6.0)	4 (4.0)	
Anastomotic leak (postop)	Absent	287 (96.3)	98 (99.0)	0.308 ***
	Present	11 (3.7)	1 (1.01)	
Intraabdominal abscess (postop)	Absent	285 (95.64)	94 (95.0)	0.782 ***
	Present	13 (4.4)	5 (5.1)	
Pulmonary complications (postop)	Absent	283 (95.0)	92 (92.9)	0.607 **
	Present	15 (5.0)	7 (7.1)	
Wound infection (postop)	Absent	265 (88.9)	81 (81.8)	0.097 **
	Present	33 (11.1)	18 (18.2)	
Sepsis (postop)	Absent	288 (96.6)	88 (88.9)	0.006 **
	Present	10 (3.4)	11 (11.1)	
Metabolic complications (postop)	Absent	294 (98.7)	89 (89.9)	<0.001 ***
	Present	4 (1.3)	10 (10.1)	
Neoadjuvant therapy	Absent	244 (83.6)	76 (80.9)	0.653 **
	Present	48 (16.4)	18 (19.2)	
Adjuvant CT	Absent	74 (26.6)	43 (45.7)	0.001 *
	Present	204 (73.4)	51 (54.3)	
Adjuvant RT	Absent	246 (88.5)	86 (91.5)	0.536
	Present	32 (11.5)	8 (8.5)	

ASA: American Society of Anesthesiologists, CT: Chemotherapy, DM: Diabetes Mellitus, HT: Hypertension, Preop: Preoperative, Postop: Postoperative, RT: Radiotherapy, *: Pearson chi square test; **: Chi-square test with Yates correction ***: Fisher’s exact chi-square test.

**Table 8:** Multivariate analysis of factors associated with mortality (backward stepwise regression).

Factors	B	SE	Wald	Sig.	Exp (B)	95% CI	
						Lower	Upper
Positive LN retrieved	0.129	0.037	11.89	0.001	1.14	1.06	1.224
Differentiation (Moderate)	1.098	0.542	4.10	0.043	2.99	1.04	8.69
Differentiation (Poorly)	1.518	0.666	5.20	0.023	4.57	1.24	16.83
Differentiation (Undifferentiated)	1.938	0.881	4.84	0.028	6.95	1.24	39.05
Intestinal obstruction (preop)	0.981	0.366	7.18	0.007	2.67	1.30	5.47
Intestinal perforation (preop)	2.465	0.582	17.95	<0.001	11.76	3.76	36.79
Metastasis	1.049	0.393	7.14	0.008	2.86	1.32	6.16

LN: Lymph node, Preop: Preoperative, Postop: Postoperative, Hosmer and Lemeshow test (Chi-square: 9.30; *p* = 0.315).

**Table 9:** Comparison of continuous variables between non-metastatic and metastatic CRC subgroups.

Parameters [Median (95%CI)]	Non-Metastatic	Metastatic	*p* *
From diagnosis to surgery (days)	8 (8–10)	8 (7–10)	0.490
From surgery to pathology (days)	21 (19–24)	22 (20–31)	0.215
Age (years)	64 (63–66)	59 (54–65)	0.001
Preoperative CA19.9	22 (18–34)	30 (16–82)	0.360
Preoperative CEA	2 (2–3)	6 (4–16)	<0.001
Tumor size (mm)	45 (45–50)	50 (47–56)	0.054
Total LN retrieved (number)	22 (21–25)	25 (22–29)	0.598
Positive LN retrieved (number)	0 (0–1)	3 (2–5)	<0.001
Hospital stay (days)	8 (8–10)	12 (10–15)	<0.001
Follow up (months)	50 (48–53)	44 (34–50)	0.002

CA 19-9: Carbohydrate antigen 19-9, CEA: Carcinoembryonic antigen, CI: Confidence interval, IQR: Interquartile range, LN: Lymph node, * Mann–Whitney U test.

**Table 10:** Comparison of categorical variables between non-metastatic and metastatic CRC subgroups.

Parameters	Categories	Non-Metastatic	Metastatic	*p*
Groups	Pre-COVID-19	176 (55.5)	37 (46.3)	0.137 *
	COVID-19 Era	141 (44.5)	43 (53.8)	
Gender	Male	187 (59.0)	48 (60.0)	0.870 *
	Female	130 (41.0)	32 (40.0)	
Overall comorbidity	Absent	159 (50.2)	48 (60.0)	0.115 *
	Present	158 (49.8)	32 (40.0)	
DM	Absent	246 (77.6)	71 (88.8)	0.039 **
	Present	71 (22.4)	9 (11.3)	
HT	Absent	230 (72.6)	65 (81.3)	0.148 **
	Present	87 (27.4)	15 (18.8)	
Pulmonary	Absent	286 (90.2)	73 (91.3)	0.947 **
	Present	31 (9.8)	7 (8.8)	
Cardiac	Absent	266 (83.9)	65 (81.3)	0.687 **
	Present	51 (16.1)	15 (18.8)	
Thyroid	Absent	288 (90.9)	77 (96.3)	0.175 **
	Present	29 (9.1)	3 (9.4)	
ASA	ASA I	14 (4.4)	6 (7.5)	0.709 *
	ASA II	134 (42.3)	33 (41.3)	
	ASA III	163 (51.4)	40 (50.0)	
	ASA IV	6 (1.9)	1 (1.3)	
Tumor locations	Transverse Colon	5 (1.6)	1 (1.39)	0.838 *
	Sigmoid	62 (19.6)	17 (21.3)	
	Right Colon	70 (22.1)	15 (18.8)	
	Rectum	113 (35.6)	24 (30.0)	
	Rectosigmoid	13 (4.1)	5 (6.3)	
	Left Colon	24 (7.6)	8 (10.0)	
	Cecum	30 (9.5)	10 (12.5)	
Timing of surgery	Elective	244 (77.0)	57 (71.3)	0.357 **
	Emergency	73 (23.0)	23 (28.7)	
Intestinal obstruction (preop)	Absent	256 (80.8)	64 (80.0)	1.000 **
	Present	61 (19.2)	16 (20.0)	
Intestinal perforation (preop)	Absent	305 (96.2)	73 (91.3)	0.078 ***
	Present	12 (3.8)	7 (8.8)	
Type of surgery	Open	140 (44.2)	65 (81.3)	<0.001 *
	Laparoscopic	166 (52.4)	11 (13.8)	
	Conversion	11 (3.5)	4 (5.0)	
Ostomy (during index surgery)	Absent	165 (52.1)	38 (47.5)	0.529 *
	Present	152 (47.9)	42 (52.5)	
Mucinous component	Absent	274 (88.1)	63 (82.9)	0.306 **
	Present	37 (11.9)	13 (17.1)	
Tumor differentiation	Well	74 (23.9)	7 (9.2)	0.001 *
	Moderately	195 (62.9)	52 (68.4)	
	Poor	31 (10.0)	17 (22.4)	
	Undifferentiated	10 (3.2)	0 (0.0)	
Primary tumor (T)	T1	15 (4.8)	0 (80.0)	<0.001 *
	T2	45 (14.4)	2 (2.5)	
	T3	205 (65.5)	25 (31.6)	
	T4	44 (14.1)	52 (65.8)	
	Tis	4 (1.3)	0 (0.0)	
Lymph node involvement (N)	N0	180 (57.5)	15 (18.8)	<0.001 *
	N1	84 (26.8)	26 (32.5)	
	N2	49 (15.7)	38 (47.5)	
	N3	0 (0.0)	1 (1.3)	
Perineural invasion	Absent	221 (70.6)	25 (32.5)	<0.001 *
	Present	92 (29.4)	52 (67.5)	
Lymphovascular invasion	Absent	148 (47.3)	8 (10.1)	<0.001 *
	Present	165 (52.7)	71 (89.9)	
Overall complications (postop)	Absent	236 (74.4)	38 (47.5)	<0.001 **
	Present	81 (25.6)	42 (52.5)	
Intestinal obstruction (postop)	Absent	302 (95.3)	73 (91.3)	0.173 ***
	Present	15 (4.7)	7 (8.8)	
Anastomotic leak (postop)	Absent	307 (96.8)	78 (97.5)	1.000 ***
	Present	10 (3.2)	2 (2.5)	
Intraabdominal abscess (postop)	Absent	306 (96.5)	73 (91.3)	0.065 ***
	Present	11 (3.5)	7 (8.8)	
Pulmonary complications (postop)	Absent	302 (95.3)	73 (91.3)	0.173 ***
	Present	15 (4.7)	7 (8.8)	
Wound infection (postop)	Absent	284 (89.6)	62 (77.5)	0.007 **
	Present	33 (10.4)	18 (22.5)	
Sepsis (postop)	Absent	304 (95.9)	72 (90.0)	0.048 ***
	Present	13 (4.1)	8 (10.0)	
Metabolic complications (postop)	Absent	307 (96.8)	76 (95.0)	0.494 ***
	Present	10 (3.2)	4 (5.0)	
Neoadjuvant therapy	Absent	253 (82.1)	67 (85.9)	0536 **
	Present	55 (17.9)	11 (14.1)	
Outcomes	Alive	254 (80.1)	44 (55.0)	<0.001 **
	Dead	63 (19.9)	36 (45.0)	

ASA: American Society of Anesthesiologists, CT: Chemotherapy, DM: Diabetes Mellitus, HT: Hypertension, Preop: Preoperative, Postop: Postoperative, RT: Radiotherapy, *: Pearson chi square test; **: Chi-square test with Yates correction ***: Fisher’s exact chi-square test.

**Table 11:** Multivariate analysis of factors associated with metastasis (backward stepwise regression).

Factors	B	SE	Wald	Sig.	Exp (B)	95% CI	
						Lower	Upper
Preop CEA	0.018	0.01	9.47	0.002	1.02	1.01	1.05
Lymph node involvement	1.583	0.49	10.1	0.002	4.87	1.83	12.95
Perineural invasion	0.778	0.36	4.55	0.033	2.17	1.07	4.45

Preop: Preoperative, CEA: Carcinoembryonic antigen, Hosmer and Lemeshow test (Chi-square: 4.47; *p* = 0.812).

## Data Availability

The datasets analyzed during the current study are available from the corresponding author on reasonable request.
